# The burden of legionnaires’ disease in Belgium, 2013 to 2017

**DOI:** 10.1186/s13690-020-00470-7

**Published:** 2020-10-07

**Authors:** Christina Fastl, Brecht Devleesschauwer, Dieter van Cauteren, Adrien Lajot, Mathias Leroy, Valeska Laisnez, Carole Schirvel, Romain Mahieu, Denis Pierard, Charlotte Michel, Stéphanie Jacquinet

**Affiliations:** 1 Student of the Master of Science Program in Public Health, University of Southern Denmark, Esbjerg, Denmark; 2 Epidemiology of Infectious Diseases, Department of Epidemiology and Public Health, Sciensano, Sciensano, Rue J Wytsman 14, 1050 Brussels, Belgium; 3 Department of Veterinary Public Health and Food Safety, Ghent University, Merelbeke, Belgium; 4 Lifestyle and Chronic Diseases, Department of Epidemiology and Public Health, Sciensano, Brussels, Belgium; 5 Agency for Care and Health, Infection Prevention and Control, Flemish Community, Brussels, Belgium; 6 Agence pour une vie de qualité, Infection Prevention and Control, Wallonia, Charleroi, Belgium; 7 Common Community Commission, Infection Prevention and Control, Brussels, Belgium; 8 Vrije Universiteit Brussel, Universitair Ziekenhuis Brussel, National Reference Center for Legionella, Brussels, Belgium; 9 Laboratoire Hospitalier Universitaire de Bruxelles (LHUB-ULB), National Reference Center for Legionella, Brussels, Belgium

**Keywords:** Legionnaires’ disease, *Legionella*, Incidence, Disability-adjusted life years (DALYs), Burden of disease

## Abstract

**Background:**

Legionnaires’ disease (LD) is a severe bacterial infection causing pneumonia. Surveillance commonly underestimates the true incidence as not all cases are laboratory confirmed and reported to public health authorities. The aim of this study was to present indicators for the impact of LD in Belgium between 2013 and 2017 and to estimate its true burden in the Belgian population in 2017, the most recent year for which the necessary data were available.

**Methods:**

Belgian hospital discharge data, data from three infectious disease surveillance systems (mandatory notification, sentinel laboratories and the national reference center), information on reimbursed diagnostic tests from the Belgian National Institute for Health and Disability Insurance and mortality data from the Belgian statistical office were used. To arrive at an estimate of the total number of symptomatic cases in Belgium, we defined a surveillance pyramid and estimated a multiplication factor to account for LD cases not captured by surveillance. The multiplication factor was then applied to the pooled number of LD cases reported by the three surveillance systems. This estimate was the basis for our hazard- and incidence-based Disability-Adjusted Life Years (DALYs) calculation. To account for uncertainty in the estimations of the DALYs and the true incidence, we used Monte Carlo simulations with 10,000 iterations.

**Results:**

We found an average of 184 LD cases reported by Belgian hospitals annually (2013–2017), the majority of which were male (72%). The surveillance databases reported 215 LD cases per year on average, 11% of which were fatal within 90 days after diagnosis. The estimation of the true incidence in the community yielded 2674 (95% Uncertainty Interval [UI]: 2425–2965) cases in 2017. LD caused 3.05 DALYs per case (95%UI: 1.67–4.65) and 8147 (95%UI: 4453–12,426) total DALYs in Belgium in 2017, which corresponds to 71.96 (95%UI: 39.33–109.75) DALYs per 100,000 persons.

**Conclusions:**

This analysis revealed a considerable burden of LD in Belgium that is vastly underestimated by surveillance data. Comparison with other European DALY estimates underlines the impact of the used data sources and methodological approaches on burden estimates, illustrating that national burden of disease studies remain essential.

## Background

Legionnaires’ disease (LD) is a commonly underdiagnosed cause of pneumonia [1]. It is acquired through the inhalation of water droplets containing bacteria of the family Legionellaceae, which can multiply within amoeba and are ubiquitously present in various aquatic environments. There are over 50 known *Legionella* species, nearly half of which have been isolated from human samples. *Legionella pneumophila* serotype 1 accounts for more than 90% of community-acquired LD cases [2]. In Europe, the age-adjusted overall reported annual incidence of LD is 1.0 to 1.6 cases per 100,000 persons but varies substantially between countries, which is likely influenced by their LD detection and reporting capabilities [3, 4]. The disease predominantly affects males and persons aged above 65 years and is fatal in around 9% of reported cases [3]. Most reported cases occur sporadically and are community-acquired [3]. LD cannot be clinically distinguished from pneumonia caused by other agents; a definite diagnosis therefore requires the confirmation of the pathogen in specimen of the patient. This necessity of specific diagnostic tests and their shortcomings have been suggested to contribute to its frequent underreporting by surveillance systems, along with lacking awareness for the disease by health professionals [5]. Previous studies investigating the burden of LD have therefore included means to account for cases missed by surveillance in their analysis [6, 7].

The objectives of this study were to present indicators that reflect the impact of LD in Belgium between 2013 and 2017 and to estimate the true burden of the disease in the specific context of the Belgian population in 2017.

## Methods

### Reference period and population

Different data sources were used to describe and estimate the burden of LD in the Belgian population. We summarized information on the reported cases, hospitalizations and deaths due to LD for all years between 2013 and 2017 for which the respective data were available. For the year 2017, we also estimated the true incidence of LD in Belgium and calculated the Disability Adjusted Life Years (DALYs) caused by the disease. This was only done for 2017, as it was the only year with fully available data for this purpose after 2016, the year in which the reimbursement of the urinary antigen (UAg) test was introduced in Belgian hospitals (as described below), which is believed to have affected the LD detection frequency in the country. Incidence and DALY rates per 100,000 population were calculated based on the Belgian population on the 1st of January 2017 [8].

This article does not address Pontiac fever, another clinical manifestation of a *Legionella* spp. infection that only has a mild, flu-like course and usually does not require treatment, as it is not included in the current case definition for LD by the European Union [9].

### Data sources

#### Hospital discharge data

The hospital discharge data (HDD) are a national and compulsory database managed by the Belgian Federal Public Service Health, Food chain safety and Environment. Since 1991, all hospitals have to report data about discharge diagnosis, duration of stay and basic demographic characteristics of all patients. Until the end of 2014, diseases were coded according to the International Statistical Classification of Diseases and Related Health Problems, 9th revision (ICD-9). Since 2016, the 10th revision of the ICD is in use. There are no data available for 2015, the transition period.

To present the number of hospitalized LD cases, we analyzed all hospital records with a patient discharge date between 2013 and 2017 and a primary (main) or secondary diagnosis of LD (ICD-9 code: 482.84 Pneumonia due to Legionnaires’ disease; ICD-10 code: A48.1, Legionnaires disease).

Furthermore, as part of the estimation of the true incidence of LD in Belgium in 2017 (described below), hospital records with bacterial pneumonia as primary or secondary diagnosis (ICD-10 codes J13 and J15–J18) from 2017 were extracted and used to estimate the proportion of LD cases that were not tested for *Legionella* spp. and therefore not captured by surveillance.

#### Belgian surveillance systems for legionnaires’ disease

There are three Belgian surveillance systems that register diagnosed cases of LD; i.e., mandatory notification (MN), sentinel laboratories (SL) and the national reference center (NRC). The surveillance system of SL is managed by Sciensano, the Belgian Institute of Health. Sciensano also receives data from the NRC and MN each year in order to carry out its missions of surveilling infectious diseases and providing data to the World Health Organization (WHO) and the European Centre for Disease Prevention and Control (ECDC).

MN is a competence delegated to the regional authorities in Belgium. Doctors are required to report each confirmed case of LD (following the EU case definition of LD [9]) to the health inspectorates of one of the three regions (Flanders, Wallonia or Brussels). The health inspectorates then collect demographic information, information about the diagnostic test used and risk factors, as well as other data necessary to identify the source of infection. In 2012, a capture-recapture study (CRS) with two data sources was performed in Wallonia, which estimated a reporting completeness of 65% of the MN [10].

The SL surveillance system is based on voluntary participation by Belgian laboratories. Each participating laboratory sends weekly positive diagnoses of a series of diseases, including LD. For LD, the SL collects demographic information and reports what diagnostic test was used. All patients with a positive test for *Legionella* spp. are reported, regardless of clinical symptoms. The surveillance network is estimated to cover around 50% of the diagnostic tests performed in Belgium [11].

For LD, the NRC consists of a consortium of two laboratories that confirm the diagnoses of samples sent by clinical laboratories and perform more complex examinations (culture, PCR and typing). Cases are reported by them in accordance with the EU case definition for LD [9]. The NRC has been established in 2011 [12]. The rate of reporting completeness of this surveillance source is unknown for LD.

The data extracted from the three available data sources for this study are anonymous, but contain information about postal code, date of birth and sex of the patient. These variables were used to identify and remove duplicates between the different sources in order to calculate the unique annual number of ‘reported LD cases’ in Belgium from 2013 to 2017 that will be used in the remainder of the manuscript. To avoid mismatches, cases for which one of the three variables was missing were not considered in this process.

As part of the estimation of the true incidence of LD, the completeness of reporting of diagnosed LD cases was estimated by performing a capture-recapture study (CRS) with all three data sources for the year 2017, using a generalized linear model with a log link function and a Poisson error structure. A more detailed description of this step can be found in Additional file 1 and the outcome of the CRS is displayed in Additional file 2. We also used the data from MN Flanders to ascertain the proportions of the diagnostic tests used in Belgium (see Additional file 1), as this information was most complete from this data source.

#### Mortality data

The Belgian statistical office, Statbel, compiles demographic information and information about the causes of death of all deceased persons in Belgium. Using the variables birth date, postal code and sex, we combined this dataset with the data on reported LD cases to find cases that died within 90 days after diagnosis in each year between 2013 and 2016 (data for 2017 were not available). The threshold of 90 days was chosen as the authors believed that most deaths connected to LD would have occurred within this period and that deaths reported thereafter may have more likely been unrelated to the infection. The case fatality ratios were calculated by dividing the number of deaths we found with this approach by the total number of reported cases for each year.

#### The Belgian National Institute for health and disability insurance

The Belgian National Institute for Health and Disability Insurance (NIHDI) started reimbursing UAg tests for LD in hospitals in September 2016. For the estimation of the true incidence of LD in Belgium in 2017 (see below), we used the number of UAg tests that have been reimbursed in 2017.

More information about the institutions that provided the data can be found in Additional file 3.

### True incidence and disability-adjusted life years (DALYs)

#### True incidence of legionnaires’ disease

**Fig. 1:**
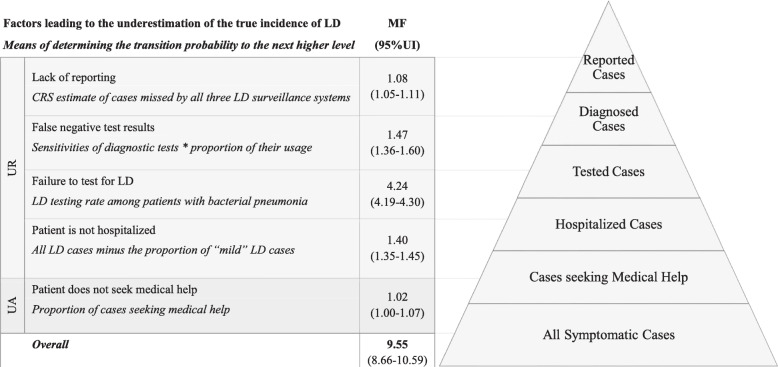
Surveillance pyramid for Legionnaire’s disease (LD) in Belgium in 2017. Illustration of how underreporting (UR; healthcare-level) and under-ascertainment (UA; community-level) affect the reported incidence of LD. The MFs account for cases lost to surveillance (reported incidence) at leach level. They were derived using this equation: MF = 1/x, where x is the probability of transitioning from one level to the next higher one we determined for the respective level based on available Belgian data and literature (see Additional file 1). MF: multiplication factor; CRS: capture-recapture study; UI: Uncertainty Interval

LD incidence was estimated using a pyramid reconstruction approach in which the different steps necessary for the reporting of a symptomatic LD infection in the community by a laboratory-based surveillance system are defined (Fig. 1). The cases captured by surveillance (top of the pyramid) are only those that seek medical care, get diagnosed correctly and are properly reported. To arrive at an estimate of all symptomatic cases in the community (bottom of the pyramid), a multiplication factor (MF) to account for cases lost to reporting at each level of the pyramid is required [13].

We assessed the probability of transitioning from the lowest to the highest level of the pyramid for 2017 by using available Belgian data and information from the literature when no Belgian data were available. Additional file 1 shows the data and data sources we used, as well as a more detailed description of the five steps in which we accounted for underreporting (at the health-care level) and under-ascertainment (at the community level) of LD.

#### Disability-adjusted life years (DALYs)

We used the DALY indicator to quantify the health burden of LD in Belgium. DALYs combine the mortality (Years of Life Lost, YLL) and morbidity (Years Lived with Disability, YLD) impact caused by a disease into a single measure, enabling the comparison of its burden between countries or regions and to other diseases. A more detailed description of the properties and calculation of this indicator can be found elsewhere [14]. To calculate the YLL, we used the reference life table from the 2017 Global Burden of Disease study which assigns a life expectancy of 87.9 years at birth to both men and women [15]. Social weighting functions were not included.

A disease model or outcome tree displays all possible manifestations of a disease that are considered in the DALY calculation. There are different methodological possibilities for constructing it. As recommended for assessing the burden of infectious diseases, we used incidence data and the hazard-based approach, where the infection constitutes the root of the outcome tree [16, 17].

We used the outcome tree previously defined in the course of the Burden of Communicable Diseases in Europe (BCoDE) project [18] and complemented it with data from the disease model from a Dutch study ([7], Online Appendix). The case fatality ratios for complicated cases and cases admitted to the intensive care unit were taken from the BCoDE outcome tree, while, in line with the Dutch study, the ratio for uncomplicated cases was set to 0%. We verified the appropriateness of these parameters in the context of this analysis by comparing their weighted average (8.5%; weighted for the respective proportions of disease severity, which are shown in Additional file 4), to the Belgium-specific case fatality ratios we estimated for the years 2013 to 2016 (see Table 1), which were of similar magnitude. The final disease model is displayed in Additional file 4.

**Table 1:** Presentation of the number of reported LD cases, the number of deaths among them and information about hospitalized LD cases in Belgium, 2013 to 2017

	2013	2014	2015	2016	2017	Mean
Reported LD cases^a^	190	186	191	227	280	215
*Deaths among reported LD cases*^*b*^	26	23	17	21	n.a.	22
*Case fatality ratio (95%CI)*	14% (9–19%)	12% (8–17%)	8.9% (5–13%)	9.3% (5–13%)	n.a.	11% (9–13%)
Hospitalized LD cases^c^	155	163	n.a.	180	232	184
*Median duration of stay in days (min–max)*	13 (0–130)	9 (0–172)	n.a.	9 (0–227)	9 (1–78)	10
*Proportion of males*	66%	79%	n.a.	71%	72%	72%
*Proportion of cases aged ≥ 65 years*	42%	40%	n.a.	41%	49%	43%

*LD* Legionnaires’ disease, *CI* Confidence interval, *ICD* International Statistical Classification of Diseases and Related Health Problems, *n.a.* No data available

^a^Combined cases from the national reference center, mandatory notification and sentinel laboratories; duplicates excluded based on overlapping birth dates, postal codes and gender

^b^Statistics Belgium death certificate data linked with data of the reported LD cases based on overlapping birth dates, postal codes and gender. Deaths within 90 days after diagnosis were included

^c^Hospitalized patients with a primary or secondary diagnosis of LD (ICD-9 code 482.84; ICD-10 code A48.1) as reported by the hospital discharge data

### Data analysis and uncertainty

To account for uncertainty in the estimation of the true incidence, we fitted a distribution to each transition probability from one level of the surveillance pyramid to the next and performed Monte Carlo simulations (10,000 iterations) to arrive at 95% uncertainty intervals (UIs) at each step of the pyramid (defined as the 2.5th and 97.5th percentile of the distribution of random values). We then multiplied the results to get an overall rate of reporting completeness and derive an overall 95% UI.

The level-specific and overall MFs, as well as their 95%UIs, were calculated as the inverses of the respective rates of reporting completeness. They are displayed next to the surveillance pyramid in Fig. 1.

To assess the robustness of the MF, we performed a variable importance analysis (sensitivity analysis) to quantify the contribution of each uncertain input variable to the overall uncertainty of the end result. Specifically, we calculated partial correlation coefficients to quantify the correlation between a given input and the output, when adjusting for all other input variables. This approach is not affected by possible interactions between input variables, in contrast to standardized regression coefficients.

Additional file 1 summarizes the model input and data sources we used to estimate the true incidence of LD in Belgium. The analyses were performed in R 3.6.1 [19], the R script can be found in Additional file 5.

The capture-recapture study (CRS) and the calculation of the DALYs were performed in SAS version 9.3 (SAS Institute Inc., Cary, NC, USA). We also used Monte Carlo simulations to account for the uncertainty in disability weights, disease durations and risks to develop the health outcomes used in the DALY calculation. The distributions we used for this step are shown in Additional file 4.

## Results

### Reported incidence, hospitalization and mortality

Table 1 displays information on LD patients reported in the community and by hospitals in Belgium and LD-associated mortality for all years with available data between 2013 and 2017. Overall, the numbers of reported and hospitalized LD cases showed increasing trends, especially after 2015. Most cases reported by hospitals were male (72%) and over 40% were aged 65 years or older. LD patients stayed in the hospitals for a median duration of 13 days in 2013 and 9 days in the years with available data thereafter, with total days ranging from 0 to up to 227 days. 11% of reported LD cases died within 90 days after diagnosis.

### True incidence

In 2017, the number of reported LD cases was 280 (2.47 reported cases per 100,000 inhabitants). The MF between cases ascertained through surveillance and cases in the community was estimated at 9.55 (95%UI: 8.66–10.59) (see Fig. 1). Thus, the estimated true incidence of LD was 2674 (95%UI: 2425–2965) cases in 2017, which corresponds to 23.62 (95%UI: 21.42–26.19) cases per 100,000 persons.

**Fig. 2:**
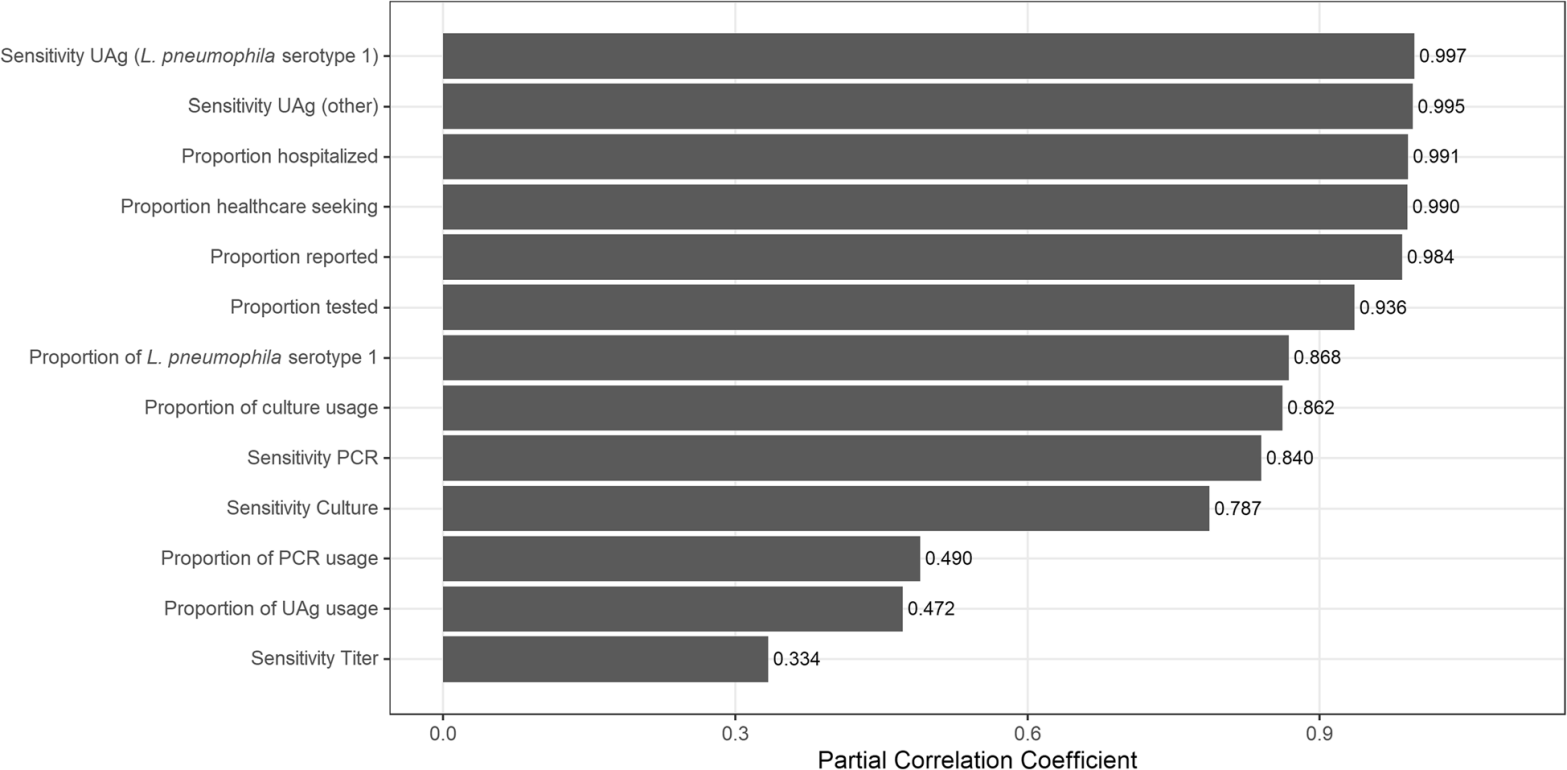
Variable Importance Analysis of the variables included in the estimation of the multiplication factor to account for underreporting and under-ascertainment of LD in Belgium in 2017. UAg: Urinary Antigen; PCR: Polymerase Chain Reaction

Figure 2 shows the results of the variable importance analysis, sorted by decreasing order of importance. The largest partial correlation coefficients were found for the test sensitivity, followed by the proportion of hospitalized LD cases, the proportion of cases that seeked healthcare, and the proportion of diagnosed cases that were reported.

### Disability-adjusted life years (DALYs)

LD caused 810 YLD (95%UI: 465–1195) and 7336 YLL (95%UI: 3988–11,231) in Belgium in 2017, amounting to 8147 (95%UI: 4453–12,426) DALYs in total or 71.96 (95%UI: 39.33–109.75) DALYs per 100,000 persons. The individual burden was 3.05 (95%UI: 1.67–4.65) DALYs per LD case, 2.74 (95%CI: 1.49–4.20) of which were attributable to YLL and 0.30 (95%CI: 0.17–0.45) to YLD.

## Discussion

This study was conducted to estimate the DALYs caused by LD in Belgium in 2017 and to present different indicators for the burden of the disease for years between 2013 and 2017, depending on the availability of data.

The surveillance data showed an increase of the reported number of LD cases after 2015. This may be connected to factors such as climate change or population ageing, which have been suggested to contribute to the generally observed rise in reported LD incidence in Europe [4]. It might, however, also be explained by a rising awareness for the disease among health professionals and improved access to the UAg test following the introduction of its reimbursement in Belgian hospitals in September of 2016.

In line with previous observations [3, 4], persons with male sex were predominantly affected by LD (72%). This sexual dimorphism has also been reported for most other infections of the lower respiratory tract and has been suggested to be to be related biological factors, such as differing levels of sex hormones, which are known to impact immune activation. Women, have, for example, higher levels of the immune-stimulating sex hormone estradiol than men while they produce less testosterone, which has anti-inflammatory properties. Factors, such as anatomical differences or differences in lifestyles and socioeconomic statuses between the sexes, may also contribute to their unequal vulnerability to lower respiratory infections [20, 21].

Our DALY calculation revealed a considerable burden which was mainly due to premature mortality, with 3.05 DALYs per case (95%UI: 1.67–4.65) and 8147 (95%UI: 4453–12,426) total DALYs per year, corresponding to 71.96 (95%UI: 39.33–109.75) DALYs per 100,000 population in 2017. To put this result into perspective, the total burden of foodborne diseases in European countries with a low mortality, such as Belgium, was estimated at 41 DALYs per 100,000 population in 2010 [22].

There are two other European studies that also estimated the true incidence of LD in the community and used it for DALY calculations. One assessed the burden of infectious diseases in the countries of the European Union (EU) and the European Economic Area (EEA) from 2009 to 2013 in the frame of the BCoDE project, and the other one estimated the burden specifically for the Netherlands from 2007 to 2011 [6, 7].

Their results only partly agree with our estimates. Estimates of DALYs per case are largely determined by the parameters of the disease model. The proportion of fatal cases is especially influential for acute and severe diseases such as LD. As our outcome tree was very similar to the one used by the BCoDE study, our DALYs per case estimate of 3.05 was comparable to their estimate of 3.04 DALYs per case. The Dutch study, however, assumed a higher proportion of non-hospitalized cases with a negligible risk of dying, thus, their estimation of the number of DALYs per case (0.97 DALYs per case) was lower than ours. DALYs on the population-level are dependent on both the disease model and the estimated disease incidence [16]. The comparability of our results to the previous studies is therefore considerably influenced by the underlying estimation of the true incidence of LD in the population. Our estimate of the true incidence of LD was similar to the one by the Dutch study (27 cases per 100,000 population per year; rate calculated based on the mean population size of the Netherlands 2007–2011 [23]) but was around seven times higher than the estimate for the EU/EAA region by the BCoDE study (3.4 cases per 100,000 per year). As a result, our estimation of the population-level DALYs was substantially higher than in the BCoDE study (71.96 vs. 10.3 DALYs per 100,000 people per year, respectively), even though the number of DALYs per case was similar.

Our higher estimation of the population-level DALYs in Belgium in 2017 may be related to a real increase in LD incidence in Europe since the study period of the BCoDE study [4]. It is, however, important to notice that differences in methodology, data sources and assumptions have a major impact on the estimated burden of LD. The BCoDE project aimed at assessing the disease burden in Europe, thus, they pooled countries in three groups depending on the quality of their surveillance systems for which they applied three different MFs that were based on studies from France and Germany ([6], Additional file 3). We believe that by deriving our MF based on the surveillance pyramid, utilizing specific Belgian data and information from the literature, our estimate most appropriately approximates the situation in this country. Our results are strengthened by their compliance with previous studies investigating the rate of LD among persons with community-acquired pneumonia (CAP), such as the German CAPNEZ study, which found that 3.8% of CAP cases were attributable to *Legionella* spp. infections [24]. When compared to the number of pneumonia cases reported by the HDD, our estimated true incidence of LD accounted for around 3.3% of them.

Nevertheless, our assessment of the true incidence of LD has several limitations.

First, one of the surveillance databases we accessed to ascertain the number of reported LD cases in 2017, the SL, does not include pneumonia in the definition of LD cases, but reports all patients who have been tested positive for *Legionella* spp., regardless of the symptoms. This may have led to an overestimation of the number of reported cases and, in turn, in the estimated true incidence of LD. However, given that pneumonia is included in the official case definition of LD by the EU [9], physicians usually only order a test for *Legionella* spp. if the patient presents with this symptom. Furthermore, only 16% of reported cases were solely reported by the SL and not by one of the two other surveillance databases (see Additional file 2). Therefore, this limitation is not expected to have influenced the results greatly.

Second, in absence of more detailed information on the clinical picture of pneumonia patients in Belgium, we assumed that patients suffering from LD have the same chance of being tested for *Legionella* spp. as all patients hospitalized with bacterial pneumonia (see Additional file 1). While, in theory, pneumonia is the only clinical criterion for a LD diagnosis [9], the testing rate among actual LD patients might still be higher than among bacterial pneumonia cases. This may have led to an overestimation of the incidence of LD at the community level, especially considering that this step contributed most to the overall estimated incidence (44% of overall MF).

Third, the HDD is an administrative tool built for financial purposes and does not collect data for epidemiological use. The diagnosis accuracy and the consistency in disease coding may therefore be questioned. Using this data to estimate the testing rate for *Legionella* spp. might have led to an over- or underestimation of the true incidence of LD.

Fourth, we assumed that all cases of LD reported to one of the Belgian surveillance databases were hospitalized. This hypothesis was supported by different Belgian experts (health inspectors, NRC) and by data from MN in Flanders but since the information was not available from all sources, it is possible that some of the reported LD cases were diagnosed outside of hospitals. In this case, the MF to account for cases not hospitalized may have been overestimated while the MF to adjust for cases not tested for *Legionella* spp. might have been too low, considering that the reimbursement of the UAg only applies to hospitals.

Finally, as we did not have information on the hospitalization rate among LD patients in Belgium, we used the rate defined in the outcome tree of the BCoDE project as an approximation even though it might not completely apply to the Belgian context.

The results of the variable importance analysis for the variables included in the estimation of the true incidence of LD indicated that the sensitivity of the UAg test, the proportion of hospitalized LD cases, the proportion of cases seeking medical help and the reporting completeness of diagnosed cases had the largest influence on the overall uncertainty of the total MF (see Fig. 2). Future research should focus on these elements to reduce uncertainties and obtain more precise LD burden estimates.

The DALY calculation itself was strengthened by our usage of the outcome tree previously defined in the course of the BCoDE project, which we complemented with additional parameters from the disease model of the Dutch study. Furthermore, to validate the case fatality ratios defined in this outcome tree, which were highly influential on the burden estimation, we compared them to the Belgium-specific case fatality ratio we estimated for the years 2013 to 2016 based on LD surveillance and overall mortality data (see Table 1). This validation constitutes another strength of the DALY calculation. A limitation was, however, that we applied the same disease durations and case fatality ratios to both genders and all age groups, even though in reality differences are to be expected. This might have led to an overestimation of the YLL.

Further research is necessary to overcome these data gaps as they may have an important impact on the final DALY estimations.

## Conclusion

This study was the first assessment of the true burden of LD in Belgium. We found LD to cause a substantial health burden in Belgium that is highly underestimated by existing surveillance systems that are based on laboratory confirmed cases. The differences between our results and the outcomes of the BCoDE study illustrate the impact of the used data sources and methodological approaches on the final estimates. Therefore, national burden of disease studies remain an essential complement to international studies.

## Supplementary information

**Additional file 1.** Model input and description of the steps to account for the underestimation of the true incidence of Legionnaires’ disease in Belgium, 2017.

**Additional file 2.** Results of the Capture-Recapture Study.

**Additional file 3.** Additional information about the data sources used.

**Additional file 4.** Outcome tree for Legionnaires’ disease in Belgium.

**Additional file 5.** R Script of the Monte Carlo simulations to account for uncertainty in the estimation of the true burden of Legionnaires’ disease in Belgium, 2017.

## Data Availability

The incidence, mortality and hospitalization data that were used in this study are considered third party data and are not publicly available. Data are however available from the authors upon reasonable request and with permission of the respective data provider. The data that were used to calculate the multiplication factor (MF), the R script of the uncertainty estimation for the MF and the model input for the DALY calculation are displayed in the Additional files.

## Abbreviations

BCoDE study: Burden of Communicable Diseases in Europe study
CAP: Community-Acquired Pneumonia
CI: Confidence Interval
CRS: Capture-Recapture Study
DALYs: Disability-Adjusted Life Years
ECDC: European Centre for Disease Prevention and Control
EU: European Union
EEA: European Economic Area
HDD: Hospital Discharge Data
ICD: International Statistical Classification of Diseases and Related Health Problems
LD: Legionnaires’ Disease
MF: Multiplication Factor
MN: Mandatory Notification
NIHDI: National Institute for Health and Disability Insurance
NRC: National Reference Centre
SL: Sentinel Laboratories
UA: Under-ascertainment
UAg: Urinary Antigen
UI: Uncertainty Interval
UR: Underreporting
WHO: World Health Organization
YLD: Years Lived with Disability
YLL: Years of Life Lost
