# Supplementary material for: The burden of legionnaires’ disease in Belgium, 2013 to 2017
Source: Arch Public Health. 2020 Oct 7;78:92. doi: 10.1186/s13690-020-00470-7 (PMC7539445; doi:10.1186/s13690-020-00470-7)
Supplement: Supplementary file 1 — Additional file 1. Model input and description of the steps to account for the underestimation of the true incidence of Legionnaires’ disease in Belgium, 2017. [file 13690_2020_470_MOESM1_ESM.pdf]

Additional file 1

Model input for the estimation of the true incidence of Legionnaires' disease (LD) in Belgium, 2017.

**Model input**		**Data Sources**	**Distribution**	**Data for model input**
Legionnaires' disease – reported cases		Annual number of cases of *Legionella* spp. reported by the Sentinel Laboratories (SL), National Reference Center (NRC) and Mandatory Notification (MN) in 2017 (combined based on birthdates, postal codes and gender, duplicates deleted).	-	SL, NRC & MN data (2017)
Account for underreporting	Account for lack of reporting of diagnosed cases	Completeness of case reporting to SL, NRC and MN in 2017, estimated by performing a capture-recapture study with all three data sources.	Beta[2]	0.93; 95%CI: 0.90,0.95
	Account for false negatives[1]	Sensitivities of the available diagnostic tests for *Legionella* spp. and the proportion of their usage in Belgium (in line with the experience of the University Hospital Brussels).		
	Sensitivities of diagnostic tests	Sensitivity of Urinary antigen (UAg) test (1)		
		L. pneumophila serotype 1	Beta[2]	0.74; 95%CI: 0.68,0.80
		Other	Uniform	0–0.40 (min, max)
		Proportion of serotype 1 vs. other (2)	Beta	α= 3020, β= 625
		Sensitivity of polymerase chain reaction (PCR) (3)	Beta[2]	0.97; 95%CI: 0.91,0.99
		Sensitivity of Culture (4; expert opinion[3])	PERT	0.80, 0.10–0.80 (most likely, min-max)
		Sensitivity of Titer (single) (4)	Uniform	0.40–0.60 (min, max)
	Proportion of usage in Belgium	Proportion of diagnostic tests used in MN for Flanders (reporting on diagnostic test was complete in this region) in 2017 and 2018.	Dirichlet	α= {236, 30, 6, 3} (UAg, PCR, Culture, Titer)

	Account for cases not tested for LD	Tested: Number of reimbursed UAg tests in 2017 (source: National Institute for Health and Disability Insurance) times proportion of UAg usage in Belgium (86%; derived from MN Flanders data, see step above);<br>Not tested: bacterial pneumonia hospitalization records from 2017 (source: Belgian Hospital Discharge Data; ICD-10 codes J13 and J15–J18) minus number of tested cases.	Beta	α= 17804, β=57705 (tested, not tested)
	Account for LD cases not hospitalized	Proportion of hospitalizations among LD cases, estimate taken from BCoDE project outcome tree (5).	Beta[2]	0.72; 95%CI: 0.69,0.74
Account for under-ascertainment		LD is a severe illness, therefore (nearly) all symptomatic cases will seek medical help (expert opinion[4]).	Beta[2]	1 (most likely); 95%CI: 0.95,1)

[1]Since all diagnostic tests were highly specific (UAg: 99% (1); PCR: 99% (3); culture: 100%; titer: >95% (4)), we only accounted for false negative, but not for false positive test results.

[2]We used the betaExpert function from the prevalence package to fit a Beta distribution to the mean values and their 95%CIs. The fitted values can be seen in the R code in Additional file 5.

[3]Expert opinion by a microbiologist from a Belgian national reference center.

[4]Expert opinion by a Belgian national medical epidemiologist and regional Belgian health inspectors.

Steps to account for the underestimation of the true incidence of LD by surveillance systems in Belgium in 2017

Underreporting (UR)

We accounted for UR in four steps. Firstly, we performed a capture-recapture study (CRS) with data on all reported LD cases from the three Belgian LD surveillance databases. The CRS has its origin in biological studies on wild animal populations. It is used to generate an estimate of the true population size by assessing the completeness of population samples from different sources based on their overlaps (6). We identified overlaps between the surveillance databases based on the variables birth date, postal code and gender. The CRS output was used to arrive at a MF accounting for diagnosed cases that have not been correctly reported to either of them. In the second step, we accounted for LD cases that were tested for a *Legionella* spp. infection but received a false negative result. To do so, we identified the sensitivities of the available LD testing methods from the literature (1-4) and derived the proportions of their usage in Belgium from the Mandatory Notification database in Flanders. This database was believed to be most representative for the general situation in Belgium. We calculated the sum of the products of the sensitivities and respective proportions of usage to arrive at an estimate of the proportion of tested LD cases, who received a correct positive testing result.

The third step was to assess the number of cases that were hospitalized, but not tested for a *Legionella* spp. infection. Here we determined the proportion of persons who were tested for *Legionella* spp. among all Belgian patients hospitalized with a bacterial pneumonia infection in 2017. We retrieved the number of primary and secondary diagnosis of bacterial pneumonia from the HDD (ICD-10 codes J13 and J15-J18). The number of diagnostic tests used was estimated by multiplying the number of reimbursed urinary antigen tests performed in Belgian hospitals in 2017, as reported by the NIHDI (National Institute for Health and Disability

Insurance), with its proportion of usage to diagnose LD compared to other diagnostic tools (86%). Since pneumonia is the only clinical criterion for a LD diagnosis (7), we assumed that the overall testing rate among bacterial pneumonia patients could be a proxy for the rate among actual LD cases. We did not include ICD-10 (or ICD-9) codes that denote a diagnosis of viral pneumonia in the analysis, as a viral infection may be distinguished from a bacterial earlier on in the diagnosis process.

Finally, we determined a multiplier to include persons that were not hospitalized but did consult a GP or other medical professional. In the BCoDE project, "mild" cases of LD were defined as those, that are less severe and don't require hospitalization. Thus, for this step, we took the proportion of "mild LD cases" from the BCoDE outcome tree (5), that we later also used for the DALY calculation.

Under-Ascertainment (UA)

Given the severity of the disease, we assumed that all or nearly all symptomatic cases will seek some form of medical help, thus the multiplier to account for UA was defined as 1 with an uncertainty range of 0.95 to 1.

References:

1. Shimada T, Noguchi Y, Jackson JL, Miyashita J, Hayashino Y, Kamiya T, et al. Systematic Review and Metaanalysis: Urinary Antigen Tests for Legionellosis. Chest. 2009;136(6):1576-85.
2. Beauté J, The European Legionnaires' Disease Surveillance N. Legionnaires' disease in Europe, 2011 to 2015. Euro Surveill. 2017;22(27):30566.
3. Avni T, Bieber A, Green H, Steinmetz T, Leibovici L, Paul M. Diagnostic Accuracy of PCR Alone and Compared to Urinary Antigen Testing for Detection of Legionella spp.: a Systematic Review. J Clin Microbiol. 2016;54(2):401-11.
4. Reller LB, Weinstein MP, Murdoch DR. Diagnosis of Legionella Infection. Clinical Infectious Diseases. 2003;36(1):64-9.
5. European Centre for Disease Prevention and Control. ECDC BCoDE toolkit [software application]. Stockholm; 2019. Available from: https://ecdc.europa.eu/en/toolkit-application-calculate-dalys.
6. Gibbons CL, Mangen M-JJ, Plass D, Havelaar AH, Brooke RJ, Kramarz P, et al. Measuring underreporting and under-ascertainment in infectious disease datasets: a comparison of methods. BMC Public Health. 2014;14(1):147.
7. European Commission. Commission Implementing Decision (EU) 2018/945 of 22 June 2018 on the communicable diseases and related special health issues to be covered by epidemiological surveillance as well as relevant case definitions (Text with EEA relevance.). Official Journal of the European Union. 2018;L 170.
