# Supplementary material for: The burden of legionnaires’ disease in Belgium, 2013 to 2017
Source: Arch Public Health. 2020 Oct 7;78:92. doi: 10.1186/s13690-020-00470-7 (PMC7539445; doi:10.1186/s13690-020-00470-7)
Supplement: Supplementary file 2 — Additional file 2. Results of the Capture-Recapture Study. [file 13690_2020_470_MOESM2_ESM.pdf]

Additional file 2

Results of the Capture-Recapture Study

Table 2.1. Results of the Capture-Recapture Study (CRS) with data from Mandatory Notification (MN), sentinel laboratories (SL) and the National Reference Center (NRC) on the incidence of Legionnaires' disease in Belgium, 2017.

Reported cases	Estimated missed cases (95% CI)	Estimated total cases (95% CI)	Mandatory Notification (MN)		Sentinel laboratories (SL)		National Reference center (NRC)	
			Cases	% of total	Cases	% of total	Cases	% of total
280	21 (15-31)	301 (295-311)	222	71-75	204	66-69	51	16-17

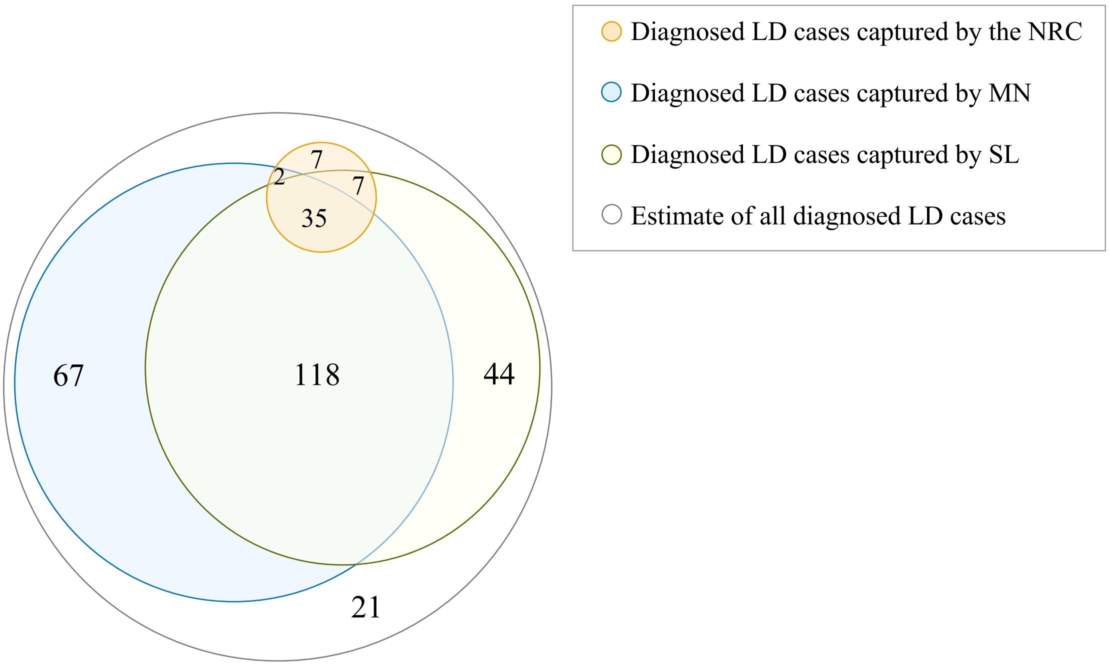

Figure 2.1. Graphic illustration of the overlaps between the cases of LD reported by the three Belgian databases that report on the incidence of the disease and the estimated cases they missed, 2017.
