# Supplementary material for: The burden of legionnaires’ disease in Belgium, 2013 to 2017
Source: Arch Public Health. 2020 Oct 7;78:92. doi: 10.1186/s13690-020-00470-7 (PMC7539445; doi:10.1186/s13690-020-00470-7)
Supplement: Supplementary file 3 — Additional file 3. Additional information about the data sources used. [file 13690_2020_470_MOESM3_ESM.pdf]

# Additional file 3:

*Additional information about the data sources used.*

| Data source | Institution providing the data | References/contact |
|---|---|---|
| Hospital Discharge Data | Federal Public Service, Health, Food Chain Safety and Environment | Direction générale des soins de santé Service Data et Information Stratégique. https://www.health.belgium.be/fr/sante/organisation-des-soins-de-sante/hopitaux/systemes-denregistrement/rhm adhoc_admDM@health.fgov.be |
| Mandatory Notification Flanders | Agenschap Zorg en Gezonheid | Meldingsplichtige infectieziekten in Vlaanderen. Richtlijnen voor de praktijk. 11/03/2019. https://www.zorg-en-gezondheid.be/sites/default/files/atoms/files/Meldingsplichtige%20infectieziekten%20in%20Vlaanderen_0.pdf |
| Mandatory Notification Wallonia | Agence pour une Vie de Qualité | Déclaration des maladies transmissibles. https://www.wiv-isp.be/matra/cf/connexion.aspx |
| Mandatory Notification Brussels | Commission Communautaire Commune | Déclaration des maladies transmissibles. https://www.ccc-ggc.brussels/fr/politique-de-la-sante/maladies-transmissibles |
| Sentinel Laboratories | Sciensano (Belgian Institute of health) | https://epidemio.wiv-isp.be/ID/Surveillance/Pages/sentinelLabs.aspx epilabo@wiv-isp.be |
| National Reference Centres | Sciensano (Belgian Institute of health) | https://epidemio.wiv-isp.be/ID/Surveillance/Pages/NRC.aspx |
| Mortality and cause of death data | The Belgian statistical office, Statbel | https://statbel.fgov.be/fr/themes/population/mortalite-et-esperance-de-vie/causes-de-deces#documents demos@economie.fgov.be |
| Reimbursement of urinary antigen test | The Belgian National Institute for Health and Disability Insurance (RIZIV/INAMI). | https://www.riziv.fgov.be/FR/nomenclature/Pages/default.aspx#NomenSoft |
