# Supplementary material for: The burden of legionnaires’ disease in Belgium, 2013 to 2017
Source: Arch Public Health. 2020 Oct 7;78:92. doi: 10.1186/s13690-020-00470-7 (PMC7539445; doi:10.1186/s13690-020-00470-7)
Supplement: Supplementary file 4 — Additional file 4. Outcome tree for Legionnaires’ disease in Belgium. [file 13690_2020_470_MOESM4_ESM.pdf]

Additional file 4:

Outcome tree for Legionnaires' disease in Belgium

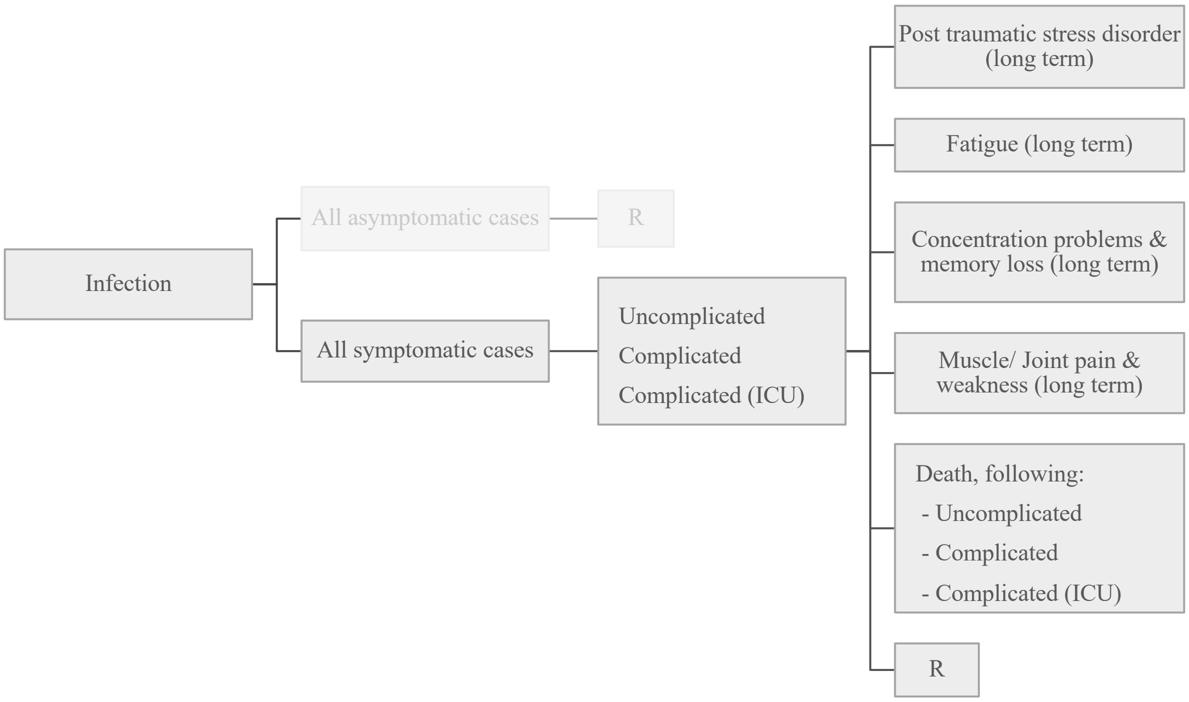

Health outcome	Risk to develop health outcome	Disability weight	Duration in years	Source
Symptomatic infection:<br>Uncomplicated<br>Complicated<br>Complicated (ICU)	 26-31%<br>47-53%<br>21-22%	 0.05 (0.04-0.06)<br>0.13 (0.1-0.15)<br>0.66 (0.58-0.73)	0.02-0.04	ECDC, 2019
Fatigue	58-81%	0.14#	1.42	van Lier et al., 2016 (Online Appendix)
Post-traumatic Stress disorder	15%	0.13#	1.42	
Concentration problems and memory loss	6.4-81%	0.14#	1.42	
Muscle joint pain and muscle weakness	25-79%	0.06#	1.42	
Death, following:<br>Uncomplicated<br>Complicated<br>Complicated (ICU)	 0%<br>5-12%<br>10-30%			
Distribution	Uniform	Pert<br>#Fixed	Uniform	

References:

1. European Centre for Disease Prevention and Control. ECDC BCoDE toolkit [software application]. Stockholm; 2019. Available from: https://ecdc.europa.eu/en/toolkit-application-calculate-dalys.
2. van Lier A, McDonald SA, Bouwknegt M, group EPI, Kretzschmar ME, Havelaar AH, et al. Disease Burden of 32 Infectious Diseases in the Netherlands, 2007-2011. PLoS One. 2016;11(4):e0153106-e.
