# Supplementary material for: The burden of legionnaires’ disease in Belgium, 2013 to 2017
Source: Arch Public Health. 2020 Oct 7;78:92. doi: 10.1186/s13690-020-00470-7 (PMC7539445; doi:10.1186/s13690-020-00470-7)
Supplement: Supplementary file 5 — Additional file 5. R Script of the Monte Carlo simulations to account for uncertainty in the estimation of the true burden of Legionnaires’ disease in Belgium, 2017. [file 13690_2020_470_MOESM5_ESM.pdf]

Additional file 5:

R Script of the Monte Carlo simulations to account for uncertainty in the estimation of the true burden of Legionnaires' disease in Belgium, 2017.

Settings

```
## required packages
library(bd)
library(fitdistrplus)
```

```
## Loading required package: MASS
```

```
## Loading required package: survival
```

```
## Loading required package: npsurv
```

```
## Loading required package: lsei
```

```
library(mc2d)
```

```
## Loading required package: mvtnorm
```

```
##
## Attaching package: 'mc2d'
```

```
## The following objects are masked from 'package:base':
##
##     pmax, pmin
```

```
library(prevalence)
```

```
## Loading required package: rjags
```

```
## Loading required package: coda
```

```
## Linked to JAGS 4.3.0
```

```
## Loaded modules: basemod,bugs
```

```
## settings
n <- 1e4
set.seed(264)
```

Define multipliers

Capture-recapture

```
(fit1 <- betaExpert(0.930, 0.900, 0.949, method = "mean"))
```

```
##      alpha     beta mean    median     mode          var      2.5%
## 1 366.8939 27.61567 0.93 0.9307261 0.932191 0.0001645978 0.9028702
##       97.5%
## 1 0.9530085
```

```
p1 <- rbeta(n, fit1$alpha, fit1$beta)
mean_ci(p1); mean_ci(1/p1)
```

```
##      mean      2.5%     97.5%
## 0.9299949 0.9027494 0.9530353
```

```
##     mean     2.5%    97.5%
## 1.075478 1.049279 1.107727
```

Test sensitivity

```
m <- rdirichlet(n, c(236, 30, 6, 3))

p21p <- rbeta(n, 3020, 625)
(fit211 <- betaExpert(0.740, 0.680, 0.800, method = "mean"))
```

```
##      alpha     beta mean    median      mode          var      2.5%
## 1 151.5573 53.24987 0.74 0.7407825 0.7423668 0.0009348556 0.6779465
##       97.5%
## 1 0.7976146
```

```
p211 <- rbeta(n, fit211$alpha, fit211$beta)
p212 <- runif(n, 0.000, 0.400)
p21 <- p21p * p211 + (1 - p21p) * p212

(fit22 <- betaExpert(0.974, 0.911, 0.992, method = "mean"))
```

```
##     alpha     beta  mean    median      mode          var      2.5%
## 1 43.0751 1.149849 0.974 0.9807461 0.9964512 0.0005599564 0.9115276
##       97.5%
## 1 0.9989839
```

```
p22 <- rbeta(n, fit22$alpha, fit22$beta)

p23 <- rpert(n, 0.100, 0.800, 0.800)
p24 <- runif(n, 0.400, 0.600)

p2 <- m[, 1] * p21 + m[, 2] * p22 + m[, 3] * p23 + m[, 4] * p24
mean_ci(p2); mean_ci(1/p2)
```

```
##      mean      2.5%     97.5%
## 0.6819914 0.6253086 0.7352415
```

```
##     mean     2.5%    97.5%
## 1.468894 1.360097 1.599210
```

Proportion tested

```
p3 <- rbeta(n, 17804.2, 75509 - 17804.2)
mean_ci(p3); mean_ci(1/p3)
```

```
##      mean      2.5%     97.5%
## 0.2357798 0.2328061 0.2387684
```

```
##     mean     2.5%    97.5%
## 4.241425 4.188158 4.295420
```

Proportion hospitalized

```
(fit4 <- betaExpert(0.715, 0.690, 0.740, method = "mean"))
```

```
##     alpha     beta  mean    median      mode          var      2.5%
## 1 894.993 356.7455 0.715 0.7151145 0.7153441 0.0001626636 0.6896831
##       97.5%
## 1 0.7396662
```

```
p4 <- rbeta(n, fit4$alpha, fit4$beta)
mean_ci(p4); mean_ci(1/p4)
```

```
##      mean      2.5%     97.5%
## 0.7147576 0.6899325 0.7396316
```

```
##     mean     2.5%    97.5%
## 1.399522 1.352024 1.449417
```

Proportion healthcare seeking

```
(fit5 <- betaExpert(best = 1, lower = 0.95))
```

```
##      alpha beta      mean    median mode          var      2.5%     97.5%
## 1 58.40398    1 0.9831661 0.988202    1 0.000273997 0.9387919 0.9995666
```

```
p5 <- rbeta(n, fit5$alpha, fit5$beta)
mean_ci(p5); mean_ci(1/p5)
```

```
##      mean      2.5%     97.5%
## 0.9827532 0.9373692 0.9995757
```

```
##     mean     2.5%    97.5%
## 1.017868 1.000424 1.066815
```

Overall multiplier

```
p <- p1 * p2 * p3 * p4 * p5

par(mfrow = c(1, 2))
hist(p)
hist(1/p)
```

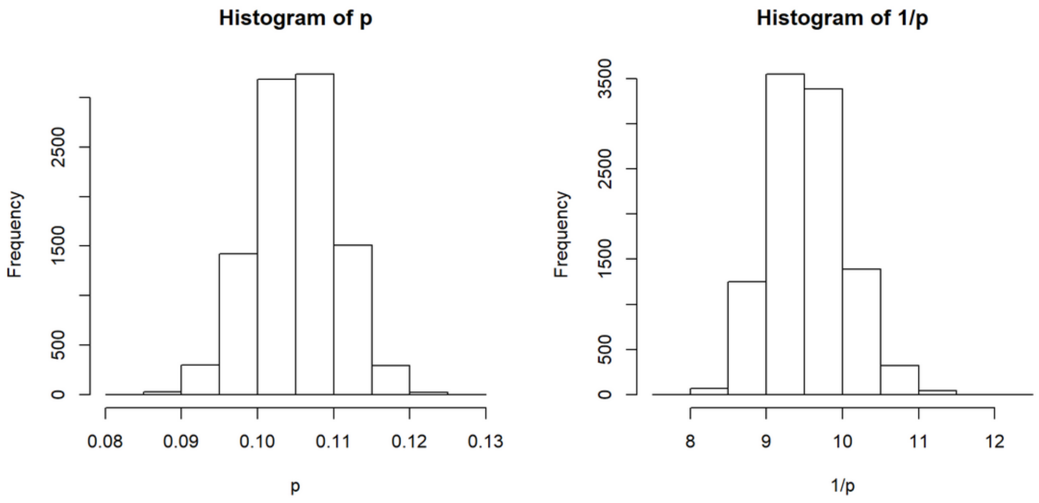


```
mean_ci(p)
```

```
##       mean       2.5%      97.5%
## 0.10504465 0.09438897 0.11544347
```

```
mean_ci(1/p)
```

```
##      mean      2.5%     97.5%
##  9.545060  8.662248 10.594458
```

Export samples

```
write.csv(
  1/p,
  row.names = FALSE,
  file = "mf-legionella-v3.csv")
```

Fit distribution

```
(fit <- fitdist(p, "beta"))
```

```
## Fitting of the distribution ' beta ' by maximum likelihood
## Parameters:
##          estimate Std. Error
## shape1   338.6464   4.780436
## shape2  2885.1950  40.754766
```

```
plot(fit)
```

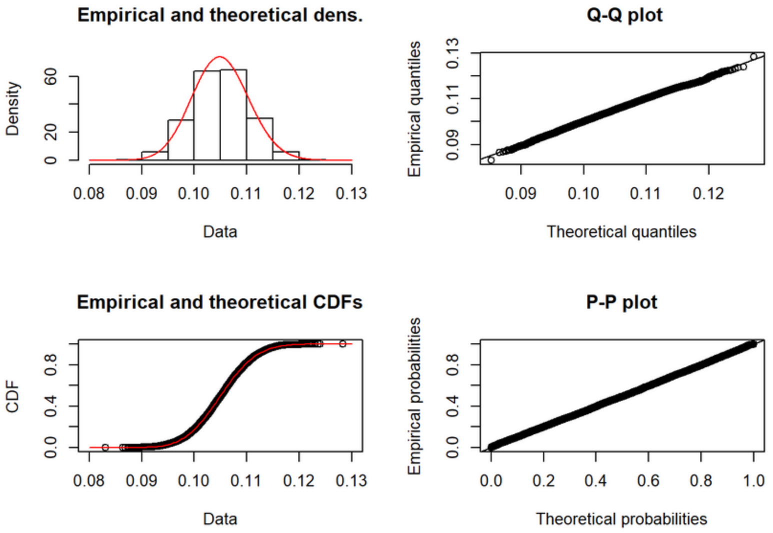


```
## compare MF estimates
mean_ci(1 / rbeta(n, fit$estimate["shape1"], fit$estimate["shape2"]))
```

```
##      mean      2.5%     97.5%
##  9.536374  8.624212 10.542949
```

```
mean_ci(1/p)
```

```
##      mean      2.5%     97.5%
##  9.545060  8.662248 10.594458
```

```
##rmarkdown::render("mf-legionella-v3.R")
```
